# Indoleamine 2,3-Dioxygenase (IDO) Downregulates the Cell Surface Expression of the CD4 Molecule

**DOI:** 10.3390/ijms130910863

**Published:** 2012-08-30

**Authors:** Guanyou Huang, Yaoying Zeng, Peiyan Liang, Congrong Zhou, Shuyun Zhao, Xiuyan Huang, Lingfei Wu, Xianhui He

**Affiliations:** 1 Reproductive Medicine Center, Affiliated Hospital of Guiyang Medical College, Guiyang 550004, China; E-Mails: zhcr1234@126.com (C.Z.); zhaosy68@126.com (S.Z.); 2 Institute of Tissue Transplantation and Immunology, Jinan University, Guangzhou 510632, China; E-Mails: tzengyy@jnu.edu.cn (Y.Z.); huangxiuyan@gmail.com (X.H.); thexh@jnu.edu.cn (X.H.); 3 Peking University Shenzhen Graduate School, Shenzhen 518000, China; E-Mail: victoria_lpy@hotmail.com; 4 Department of Gastroenterology, Second Affiliated Hospital, Shantou University Medical College, Shantou 515041, China; E-Mail: lingfeiwu@21cn.com

**Keywords:** human IDO, CD4, T cell, immune tolerance, immune response

## Abstract

Indoleamine 2,3-dioxygenase (IDO) has been implicated in preventing the fetus from undergoing maternal T cell-mediated immune responses, yet the mechanism underlying these kinds of IDO-mediated immune responses has not been fully elucidated. Since the CD4 molecule plays a central role in the onset and regulation of antigen-specific immune responses, and T cell is sensitive in the absence of tryptophan, we hypothesize that IDO may reduce cell surface CD4 expression. To test this hypothesis, an adenoviral vector-based construct IDO-EGFP was generated and the effect of IDO-EGFP on CD4 expression was determined on recombinant adenoviral infected C8166 and MT-2 cells, by flow cytometry and/or Western blot analysis. The results revealed a significant downregulation of cell membrane CD4 in pAd-IDOEGFP infected cells when compared to that of mock-infected cells or infection with empty vector pAd-EGFP. Further experiments disclosed that either an addition of tryptophan or IDO inhibitor could partly restore CD4 expression in pAd-IDOEGFP infected C8166 cells. Our findings suggest that downregulation of CD4 by IDO might be one of the mechanisms through which IDO regulates T cell-mediated immune responses.

## 1. Introduction

Indoleamine 2,3-dioxygenase (IDO) is predominantly expressed in parenchymal tissues such as lungs, gut, and the fetal-maternal unit during pregnancy, as well as in the male epididymis and thymus [1]. However, only some cell types constitutively express IDO, or the expression can only be detected following tissue lesion, infection, and inflammation in these tissues. These kinds of cells include trophoblast, fibroblasts, epithelial and tumor cells, tumor-associated cells, macrophages, DCs, and microglial cells in the CNS [2,3]. IDO is a cytosolic monomeric hemoprotein that catalyzes the first and rate-limiting step in the oxidative degradation of tryptophan, resulting in a number of downstream metabolites known as kynurenines [4]. Depletion of both tryptophan and kynurenines can affect the functions of T-cell [5,6]. There have been substantial interests in the role of IDO with respect to the mechanism of materno-fetal tolerance during pregnancy since Munn [7] showed that by exposing pregnant mice to 1-methyl-dl-tryptophan (1-MT), reduced IDO was able to induce a T-cell mediated rejection of allogeneic concepti, while syngeneic concepti remained intact. One subsequent study revealed that this fetal allograft rejection was accompanied by a specific inflammation characterized by T cell-dependent, antibody-independent activation of complement. In contrast, no inflammation, complement deposition or T cell infiltration was found when mice carrying syngeneic fetuses were exposed to IDO inhibitor [8]. These data demonstrate that IDO activity protects the fetus from T cell-driven local inflammatory responses to fetal alloantigens. Understanding how IDO abrogates these kinds of T cell-mediated immune responses should provide great insights into the mechanism of maternal-fetal tolerance.

Currently, two theories have been proposed to explain how IDO mediates immune tolerance. One theory is that by catabolizing tryptophan, IDO starves T cells of this essential amino acid which is indispensable for their proliferation and survival in the local microenvironment [9,10]; the other theory postulates that the downstream metabolites of tryptophan catabolism by IDO are also active in blocking T cell proliferation and inducing apoptosis [5,6]. Both tryptophan depletion and defective tryptophan catabolism are tolerogenic effectors in regulating T cell function, yet the clear mechanisms whereby they affect T cell responses remain unknown.

The CD4 molecule is a type I transmembrane glycoprotein with 58 kDa molecular weight and consists of an extracellular region with 370 amino acids, a transmembrane region with 25 amino acids and a cytoplasmic tail with 38 amino acids at the *C*-terminal end [11]. CD4 is expressed on a subset of T lymphocytes and cells of the macro-phage/monocyte lineage. Expression of CD4 on these cells is critical to the development and function of the immune system [12]. CD4 participates in forming molecular complexes involved in both T cell development and antigen recognition by T cells. CD4 interacts with nonpolymorphic regions of MHC class II molecules, and these interactions lead to increased intercellular adhesion and enhanced stimulation of T cells. A src-like tyrosine kinase, 56lck, is associated with the cytoplasmic domain of CD4 and may have a more profound influence on the whole signal cascade transduced upon encounter with antigens [13].

*In vitro* experiments show that IDO expression has a marked effect on the proliferation of bystander CD4+ T cells [14]. It has been reported that IDO mRNA expression is elevated in PBMC from HIV+ patients compared to uninfected healthy controls, and that *in vitro* inhibition of IDO with the competitive blocker 1-MT results in increased CD4+ T cells proliferative response in PBMC from HIV-infected patients [10]. Although the effects of IDO on the proliferation of CD4+ T cells is recognized, the regulation of human IDO on the CD4 molecule of the cell surface is not well characterized. Therefore, it is of special interest to know whether immunosuppressive effects of IDO are related to the expression of the CD4 molecule. Since the cell surface expression of major histocompatibility complex class I (MHC class I) antigen is suppressed in IDO genetically modified cells [15], it is possible that IDO may downregulate the expression of some other cell surface markers. An induction of IDO expression and a progressive loss of T cell function in human immunodeficiency virus type 1(HIV-1)-infected patients [10,16] raises the possibility that IDO may downregulate this cell surface molecule. This led us to hypothesize that the expression of IDO might affect the expression of CD4 on the cell surface. In view of the immunosuppressive effects of IDO, we constructed a replication—incompetent adenovirus vector expressing the human IDO gene to test the effect of IDO on CD4 expression. Our studies revealed that IDO downregulated the expression of CD4 in infected MT-2 cells or C8166 cells. Further analysis disclosed that the downregulation of CD4 expression by IDO was significantly attenuated by the addition of tryptophan or IDO inhibitor in the infected C8166 cells.

## 2. Results and Discussion

### 2.1. Infection of MT-2 Cells with Either pAd-EGFP or pAd-IDOEGFP

There is no evidence that human T cells can express IDO, however, the immuno-modulatory effects of IDO on T cells are related to the pericellular degradation of tryptophan [14]. Several cell types, including Ds and macrophages, may express IDO, which can be increased in response to IFN-γ and the expression of IDO in DCs can downregulate type 1 diabetes, in which CD4+ T cells are involved. Plasmacytoid dendritic cells (pDCs) express IDO and can downmodulate immune reactions through IDO-mediated tryptophan depletion [10]. At first, we were unable to infect PBMC with EGFP-marked adenovirus (data not shown), so we gave up using IDO-expressing DCs co-cultured with CD4+ T cells, which, after culture, could be analyzed for CD4 expression. Following this, two kinds of cell lines (MT-2 and C8166 lines) were used for the study, because the infection rate was high and (52%–74%) and the experiments were not complicated: instead of the co-culture system, the downregulation of CD4 could be checked in cultured T cell lines.

**Figure 1:**
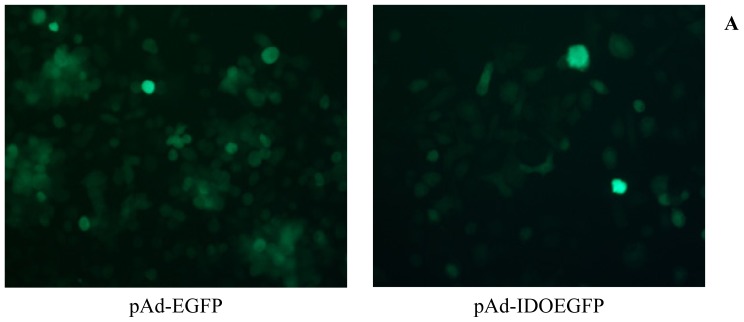

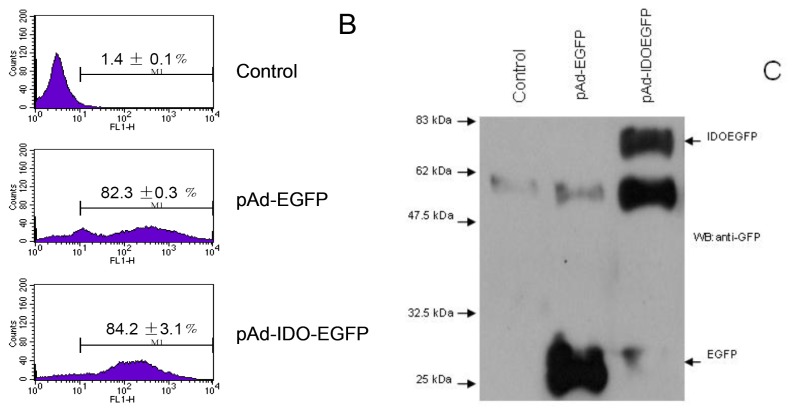
The expression of IDOEGFP in MT-2 cells. MT-2 cells were infected with either pAd-EGFP or pAd-IDOEGFP at MOI of 100 for 60 h. (**A**) After 60 h of infection, the infection was monitored by EGFP expression under fluorescent microscopy. Original magnification 300×; (**B**) The efficiency of infection was determined by flow cytometry. After 60 h of infection, the cells were harvested and the numbers of EGFP positive cells were estimated by flow cytometry. Data are shown as mean ± SD, representative of three independent experiments; (**C**) Western blot analysis of IDOEGFP expression. After 60 h of infection, the noninfected (control) and infected cells were harvested and cell lysates from about 3 × 10^5^ cells were fractionated by SDS-PAGE. IDOEGFP protein was detected using purified mouse monoclonal anti-GFP antibody at a concentration of 1:1000. EGFP = enhanced green fluorescent protein; IDO = indoleamine 2,3-dioxygenase; MOI = multiplicity of infection; pAd-EGFP = recombinant adenovirus containing EGFP gene; pAd-IDOEGFP = recombinant adenovirus containing IDOEGFP gene; SDS-PAGE = sodium dodecyl sulfate-polyacrylamide gel electrophoresis.

MT-2 cells were susceptible to the adenoviral infection. GFP expression could be visualized by fluorescent microscopy within 12 h of the addition of recombinant adenovirus (data not shown). The efficiency of infection was determined by fluorescent microscopy and flow cytometry (Figure 1A,B). As illustrated in Figure 1, more than 80% of MT-2 cells were infected by pAd-EGFP or pAd-IDOEGFP after 60 h of infection. To determine the IDOEGFP protein expression, MT-2 cells were infected with pAd-IDOEGFP at MOI of 100 for 60 h, then harvested and lysed with RIPA buffer and checked for IDOEGFP expression by Western blot using anti-GFP monoclonal antibody. As expected, EGFP (26-kDa) and IDOEGFP (68-kDa) protein bands were observed in cells infected with pAd-EGFP and pAd-IDOEGFP respectively, but not in non-infected cells (Figure 1C). To further confirm the expression of IDOEGFP fusion protein, the MT-2 cells infected with pAd-IDOEGFP were lysed and analyzed by Western blot with IDO polyclonal antibody and the results showed that a IDOEGFP protein band with a 68-kDa molecular weight was observed in these infected cells, but not in pAd-EGFP infected cells or non-infected cells (data not shown).

### 2.2. IDO Downregulates Expression of CD4

**Figure 2:**
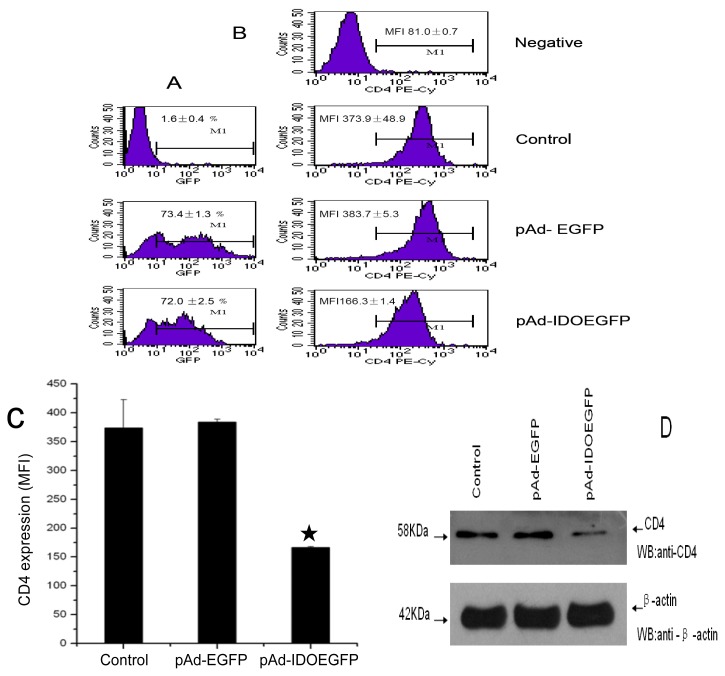
IDO downregulates CD4 protein in MT-2 cells. MT-2 cells were infected with either pAd-EGFP or pAd-IDOEGFP for 60 h. At 60 h post infection, the cells were harvested and stained with PE-conjugated anti-CD4 monoclonal antibody. FACS analysis was used to determine the efficiency of infection (**A**) and the cell surface CD4 expression (**B**,**C**) on untreated, pAd-EGFP or pAd-IDOEGFP infected MT-2 cells. Panel **A** and panel **B** illustrate the results from two-color (PE and GFP) channel analysis, whereas panel **C** depicts the quantitative analysis of CD4 expressing levels in either noninfected pAd-EGFP or pAd-IDOEGFP infected MT-2 cells. Data are shown as mean ± SD, representative of three similar experiments; (**D**) Expression of cell surface CD4 by Western blot analysis. The plasmatic membrane proteins prepared from 6 × 10^5^ noninfected cells or cell infected with either pAd-EGFP or pAd-IDOEGFP for 60 h, were fractionated by SDS electrophoresis on 8% acrylamide gel and electrotransferred onto a PVDF membrane. CD4 was detected by using mouse monoclonal anti-human CD4 antibody at a concentration of 1 μg/mL (upper line). In parallel, the cell lysates (with RIPA buffer) from the same amount of these cells were fractionated by SDS-PAGE. β-Actin was detected using rabbit polyclonal anti-β-actin antibody at a concentration of 1:300. ^★^*p* < 0.01, compared with Control and pAd-EGFP respectively.

The effect of IDOEGFP on the cell surface CD4 expression was evaluated in MT-2 cells at 60 h post infection. The CD4 expression was monitored by staining CD4 with PE-labeled antibody followed by flow cytometry analysis. As illustrated in Figure 2B,C, the expression of IDO in MT-2 cells resulted in up to 2.2-fold downregulation of cell surface CD4 compared with those of nonviral infected and mock adenoviral infected cells (mean fluorescence intensity (MFI) of 166.3 ± 1.4 *vs.* 373.9 ± 48.9, *p* < 0.01 compared with nonviral infected control for nonviral infected cells; and 166.3 ± 1.4 *vs.* 383.7 ± 5.3, *p* < 0.01 compared with mock infected control for mock adenoviral cells). To rule out the possibility that adenovirus infection and EGFP might affect CD4 expression, MT-2 cells were infected with mock recombinant adenovirus (pAd-EGFP) and the CD4 expression was analyzed. There is no significant difference in the cell surface CD4 expression between pAd-EGFP infected and non-infected MT-2 cells (MFI of 373.9 ± 48.9 *vs.* 383.7 ± 5.3, Figure 2B,C). These results suggested that the IDOEGFP expression itself specifically reduced the cell surface CD4 antigen in infected MT-2 cells. To further demonstrate that IDO downregulates the expression of CD4 on the cell surface, both the plasmatic membrane proteins and the cell lysate (lysed in RIPA buffer) extracted from infected or non-infected 6 × 10^5^ MT-2 cells were loaded onto SDS-PAGE gel, followed by Western blot with purified mouse monoclonal anti-human CD4 antibody at a concentration of 1 μg/mL, or with rabbit polyclonal anti-β-actin antibody (1:300 dilution). The results revealed that the expression of the cell surface CD4 molecule in MT-2 cells infected with pAd-IDOEGFP was reduced when compared with that in mock cells or with cells infected with pAd-EGFP (Figure 2D, the upper panel) while similar amounts of β-actin were detected in mock, pAd-EGFP or pAd-IDOEGFP infected MT-2 cells (Figure 2D, the lower panel).

### 2.3. Downregulation of the CD4 Molecule by IDO Is Partially Tryptophan Dependent

**Figure 3:**
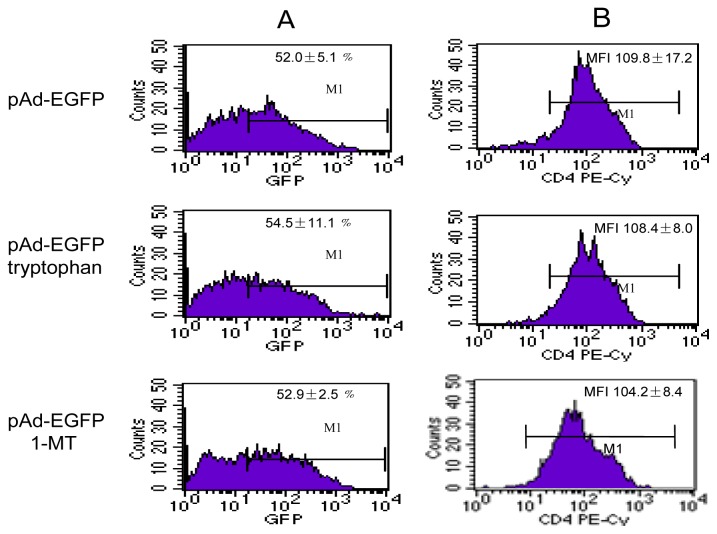

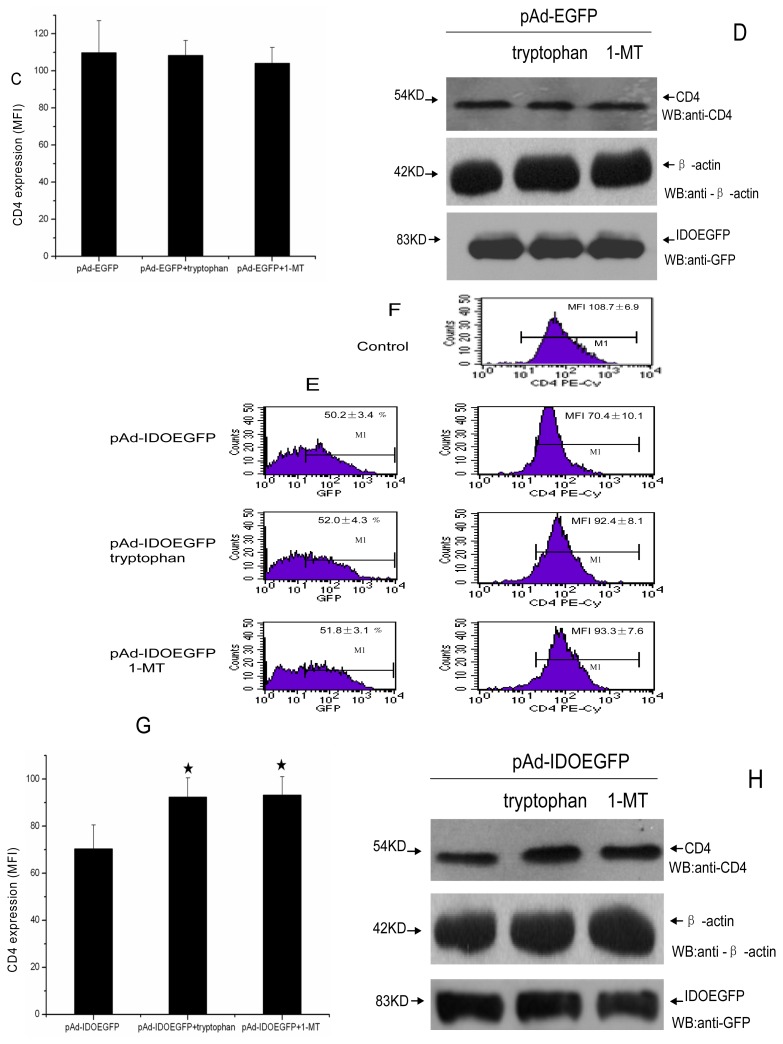
Addition of tryptophan and IDO inhibitor partially restored IDO induced downregulation of CD4 expression. C8166 cells were infected with either pAd-EGFP or pAd-IDOEGFP and added with either 200 μM tryptophan or 800 μM 1-MT at the time of infection. At 60 h post infection, the cells were harvested and stained with PE-conjugated anti-CD4 monoclonal antibody. FACS analysis was used to determine the efficiency of infection (**A**,**E**) and the cell surface CD4 expression (**B**,**C**,**F**,**G**) in untreated, pAd-EGFP or pAd-IDOEGFP infected C8166 cells added with tryptophan or 1-MT or without. Pane **A** and pane **B** illustrate the results from two-color (PE and GFP) channel analysis, whereas pane **C** depicts the quantitative analysis of CD4 expressing levels in pAd-EGFP infected C8166 cells added with or without tryptophan or 1-MT. Pane **E** and pane **F** illustrate the results from two-color (PE and GFP) channel analysis, whereas pane **G** depicts the quantitative analysis of CD4 expressing levels in pAd-IDOEGFP infected C8166 cells added with or without tryptophan or 1-MT. Data are shown as mean ± SD, representative of three similar experiments. (**D**,**H**) The expression of cell surface CD4 was carried out by Western blot analysis. The plasmatic membrane proteins prepared from C8166 cells infected with either pAd-EGFP or pAd-IDOEGFP and added with tryptophan or 1-MT or without for 60 h, were fractionated by SDS electrophoresis on 8% acrylamide gel and electrotransferred onto PVDF membrane. CD4 was detected using mouse monoclonal anti-human CD4 antibody at a concentration of 1 μg/mL in pAd-EGFP (**D**, upper line) or pAd-IDOEGFP (**H**, upper line) infected C8166 cells. In parallel, the cell lysate (with RIPA buffer) from the same amount of these cells were fractionated by SDS-PAGE. EGFP or IDOEGFP protein was detected by using purified mouse monoclonal anti-GFP antibody at a concentration of 1:1000 and β-actin was detected using rabbit polyclonal anti-β-actin antibody at a concentration of 1:300 in pAd-EGFP (pane **D**, middle and low lines) or pAd-IDOEGFP (**H**, middle and low lines) infected C8166 cells. ^★^*p* < 0.05, compared with pAd-IDOEGFP. Abbreviations: EGFP = enhanced green fluorescent protein; IDO = indoleamine 2,3-dioxygenase; MFI = mean fluorescence intensity; 1-MT = 1-methyl-dl-tryptophan; pAd-EGFP = recombinant adenovirus containing EGFP gene; pAd-IDOEGFP = recombinant adenovirus containing IDOEGFP gene; SDS-PAGE = sodium dodecyl sulfate-polyacrylamide gel electrophoresis.

To test the possible mechanism through which IDO mediates the downregulation of CD4 expression on the cell surface, l-tryptophan or IDO inhibitor 1-MT was added to C8166 cells including non-infected, pAd-EGFP and pAd-IDOEGFP infected cells at the time of infection, and at 60 h post infection, the cell surface CD4 molecule was evaluated by flow cytometry and Western blot. Addition of l-tryptophan at a concentration of 200 μM or 1-MT at a concentration of 800 μM to RPMI 1640 medium (containing 10% FBS) had no effect on the expression of CD4 in infected C8166 cells (data not shown) or pAd-EGFP infected C8166 cells (Figure 3B–D); however, the addition of tryptophan markedly increased the expression of CD4 molecule and partially restored the level of CD4 expression in pAd-IDOEGFP infected C8166 cells (MFI of 92.4 ± 8.1 *vs.* 70.4 ± 10.1, *p* < 0.05, compared with pAd-IDOEGFP infected C8166 cells, Figure 3F–H). Meanwhile, the addition of 1-MT was also able to partially restore CD4 expression in pAd-IDOEGFP infected C8166 cells (MFI of 93.3 ± 7.6 *vs.* 70.4 ± 10.1, *p* < 0.05, compared with pAd-IDOEGFP infected C8166 cells, Figure 3F–H). This partial restoration of CD4 expression in IDO infected C8166 cells by addition of tryptophan or IDO inhibitor strongly suggested that the depletion of tryptophan is involved in IDO induced downregulation of CD4 expression.

### 2.4. Effect of IDO on the Level of CD4 mRNA in C8166 Cells

**Figure 4:**
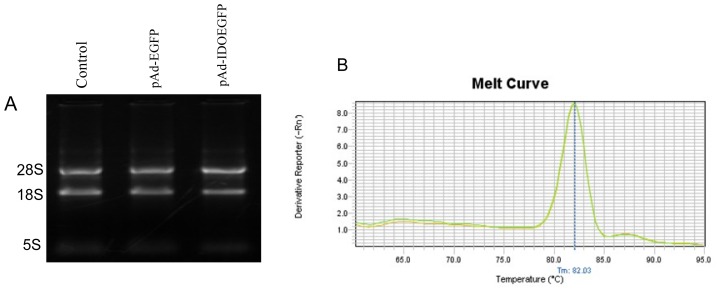
The expression of human CD4 mRNA in IDO expressing C8166 cells. C8166 cells were infected with either pAd-EGFP or pAd-IDOEGFP, and at 60 h post transfection, the total RNA was extracted and the cDNA was synthesized as described in the Materials and Methods section. Panel **A** illustrate the total RNA from untreated (control), pAd-EGFP or pAd-IDOEGFP infected C8166 cells, extracted as described in methods and subjected to electrophoresis through a native 1.5% agarose gel; Panel **B** displays melting curves of RT-PCR product generated using GeneAmp Ster One Plus SDS software.

The agarose gel electrophoresis of extracted RNA from each cell group show that the 28S, 18S, 5.8S (5S) rRNA bands were clearly visible (Figure 4A). The PCR efficiencies of CD4 gene, β-actin gene and GAPDH gene were 96%, 97.5% and 98% respectively using the method of standard curves of serial dilution of cDNA and a strong linear correlation (*R*^2^ values = 0.995) was established. The melting curves in Figure 3 show the specificity of amplification for different cell groups (Figure 4B). The primer pairs for human CD4 gene were designed as described in the Materials and Methods section. There are at least five variants of CD4 mRNA and the primer pairs are common to all the variants. However, we could not perform RT-PCR using variant analysis for the CD4 molecule, because there were no specific primer sets for each variant. The CD4 expression in the transcription level was investigated by real time PCR. We found that there was no significant difference in CD4 expression in pAd-IDOEGFP infected cells compared with either non-infected, or pAd-EGFP infected cells (Table 1). These results indicated that IDO did not affect CD4 expression at the level of mRNA.

## 3. Experimental Section

Antibodies and Chemicals—Antibodies used in Western blot, flow cytometric analysis and reverse transcriptase-polymerase chain reaction (RT-PCR) are as follows: the purified mouse monoclonal anti-GFP antibody was obtained from Clontech Inc. (USA); the purified mouse monoclonal anti-human CD4 antibody was obtained from R&D Systems (USA); the rabbit polyclonal anti-IDO antibody was obtained from Santa Cruz Biotechnology, Inc. (USA); the rabbit polyclonal anti-β-actin antibody was obtained from Beijing Boisynthesis Biotechnology Co., Ltd. (China); the polyclonal horseradish peroxidase-conjugated swine anti-rabbit immunoglobulins, and rabbit anti-mouse immunoglobulins were purchased from Dakocytomation (USA); PE-labeled anti-CD4 monoclonal antibody was purchased from BD Company (USA); the Western blot detection ECL kit was purchased from Pierce (USA); protease inhibitor cocktail, l-tryptophan, 1-MT and RIPA buffer was purchased from Sigma Aldrich (USA); ViraPower™ Adenoviral Expression System (including Gateway LR Clonase II enzyme Mix, Lipofecfamine 2000, Library Efficiency DB3.1 Competent Cells, DH5α Competent Cells, pAd/CMV/V5-DEST and pENTR 2B vectors), pEGFP-N1 vector and Trizol were purchased from Invitrogen (USA); Plasma Membrance Protein Extraction Kit was purchased from Nanjing KeyGen Biotech. Co., Ltd. (China); DMEM, RPMI 1640 and FBS were purchased from GIBCO (USA); QIAGEN OneStep RT-PCR Kits were purchased from QIAGEN (Germany).

### 3.1. Cell Culture

Cell Culture and Transfection—Human embryonic kidney 293T cells were maintained in DMEM supplemented with 10% FBS. The CD4+ C8166 T cells and CD4+ MT-2 cells were cultured in RPMI 1640 medium containing 10% FBS.

### 3.2. Adenovirus Vector Construction

The human IDO cDNAs were cut from pEGFP-IDO plasmid [17] and fused to the 5′ end of pEGFP-N1 to generate IDOEGFP. We used ViraPower™ Adenoviral Expression System (Invitrogen) to construct Adenoviral vectors carrying IDOEGFP or EGFP genes, according to the manufacturer’s instructions. The IDOEGFP fragment was generated from pEGFP-IDO plasmid, digested with *Eco*RI and *Not*I and was inserted into a pENTR™ 2B entry vector at the same sites; the EGFP fragment was generated from pEGFP-N1 plasmid digested with *Kpn*I and *Not*I and was inserted into a pENTR™ 2B entry vector at the same sites. An LR recombination reaction was performed between the cloned plasmid and pAd/CMV/V5-DEST™ destination vector by using LR Clonase™ enzyme mix, and the reaction mixture was transformed into competent DH5α bacteria, and the true expression clones were selected as ampicillin-resistant and chloramphenicol-sensitive. The success of IDOEGFP or EGFP insertion into adenoviral plasmid was confirmed by DNA sequencing. Adenoviral vectors carrying either EGFP (pAd-GFP) or IDOEGFP gene (pAd-IDOEGFP) were then linearized by *Pac*I digestion and used to transfect 293T cell using Lipofecfamine 2000 reagent. Transfected cells were monitored for GFP expression and after three cycles of freezing in a liquid nitrogen bath and rapid thawing at 37 °C, the cell lysates were used to amplify viral particles on a large scale. The viral titer was determined in a 96-well plate according to the manufacturer’s instructions.

### 3.3. Infection and GFP Detection

Recombinant adenoviruses were used to infect MT-2 cells or C8166 cells at a multiplicity of infection (MOI) of 100. Cells were harvested after 60 h of infection. The efficiency of infection was determined by flow cytometry (FACScan; Becton Dickinson Co., USA) measuring the GFP protein expression and fluorescent microscopy using a Nikon inverted microscope equipped with an FITC filter to view GFP. Images were captured by a digital camera.

### 3.4. Flow Cytometric Analysis

The expression of CD4 and the efficiency of infection on the cell surface were determined by flow cytometry. After direct immunofluorescence staining using PE-labeled anti-CD4 monoclonal antibody for 30 min, nonspecific binding was removed by washing the cells twice with cold PBS and two-color flow cytometric analyses were performed. A total of 10,000 events were collected by the FACScan, and the data were analyzed using the CellQuest software (Becton Dickinson Co.).

### 3.5. Western Blot Analysis

To detect the expression of IDOEGFP and cellular protein β-actin, the cell extracts were prepared from normal or infected cells lysed in RIPA buffer supplemented with protease inhibitor cocktail. Extracts were centrifuged at 14,000 rpm for 10 min. Then the supernatants were run onto SDS-PAGE gel and the proteins in acrylamide gel were transferred to a polyvinylidene fluoride (PVDF) membrane. Immunoblotting against IDOEGFP and β-actin was carried out. Blots were initially incubated with mouse monoclonal anti-GFP antibody at a concentration of 1:1000, or with rabbit polyclonal anti-β-actin antibody at a concentration of 1:300, followed by incubation with HRP-conjugated secondary antibody respectively. To check the expression of cell surface CD4 molecule, the plasma membrane fraction from normal or infected cells was obtained using a Plasma Membrane Protein Extraction Kit according to the manufacturer’s instructions. The plasmatic membrane proteins were fractionated by SDS electrophoresis on 8% acrylamide gel and electrotransferred onto PVDF membrane, then the membrane was incubated with 1μg/mL mouse monoclonal anti-human CD4 antibody, subsequently with HRP-conjugated rabbit anti-mouse antibody. Protein bands were visualized by using the ECL Kit followed by autoradiography.

### 3.6. Real Time PCR

Total RNA in the C8166 cells of each group was isolated by Trizol reagents (Invitrogen, USA). The purity and quantity of RNA were determined with a UV spectrophotometer with A260/A280 ratio >1.8 and the integrity of extracted RNA was tested by agarose gel electrophoresis. cDNA was generated from 3 μg of total RNA by utilizing a cDNA synthesis kit (Promega, USA) and oligo d(T) primers according to the protocol recommended by the manufacturer. The sequences of primers of human CD4 gene were designed according to the gene sequences and synthesized by Shanghai Genecore Biotechnologies, Shanghai, China. 5′ Oligonucleotide primers used for amplification were as follows: for human CD4, sense 5′-GTATGCTGGCTCTGGAAACCT-3′ anti-sense 5′-GAGACCTTTG CCTCCTTGTTC-3′; for human glyceraldehyde-3-phosphatedehydrogenase (GAPDH), sense 5′-AGAAGGCTGGGGCTCATTTG-3′ anti-sense 5′-AGGGGCCATCCACAGTCTTC-3′; for human β-actin, sense 5′-TGGCACCACACCTTCTACAATG-3′, anti-sense 5′-TCATCTTCTCGCGGTTGGC-3′. One microgram of the total cellular RNA extracted with Trizol Reagent (Invitrogen, USA) was subjected to reverse transcription by utilizing a cDNA synthesis kit (Invitrogen, USA): incubated at 70 °C for 5 min, 37 °C for 5 min, then 42 °C for 60 min, and finally cooling. The real-time quantitative RT-PCRs were performed using the ABI Step One Plus Sequence Detection System (Applied Biosystems) and analyzed with GeneAmp Ster One Plus SDS software. RT-PCR reactions were performed with the Universal TaqMan 2× PCR mastermix (Applied Biosystems, CA, USA) in a 20-μL reaction volume containing primers for human CD4 or GAPDH. The thermal cycling conditions were 2 min at 50 °C and 10 min at 95 °C, 40 cycles at 95 °C for 15 s and 60 °C for 1 min. The software was used to analyze data and calculate Ct (threshold cycle) values. The CD4 and GAPDH transcript levels were estimated using the formula 2^−ΔCt^ where ΔCt represents the difference in Ct values between target and housekeeping assays. To confirm the specificity of amplification, melting curve analysis was carried out after the last cycle of each amplification. To know the PCR efficiency the standard curves were constructed using serial dilution of cDNA from each group (each concentration in triplicate).

### 3.7. Statistics

Statistical analysis was conducted using SPSS version 10.0, and results were shown as means ± standard deviation (SD) and were analyzed statistically by using Student’s *t*-test. Probability values of <0.05 were considered significant.

## 4. Conclusions

In a set of seminal studies, Munn, Mellor *et al.* [5,8] reported evidence that IDO activity was able to prevent allogeneic fetal rejection due to IDO abrogating maternal T cell-mediated immune responses in mice. Subsequent studies have broadly extended the immunosuppressive role of IDO in a variety of physiopathological conditions, ranging from pregnancy [2,7,8,18] to transplantation [19–22], from autoimmunity [23–25] and inflammation [10,16,26,27] to neoplasia [28–30]. It is important to know how IDO modulates T cell-mediated immune responses and leads to immunosuppression, since IDO has been shown to be implicated under these physiopathological conditions. It has been reported that IDO arrested activated T-lymphocytes in the G1 phase, inhibited T-cell proliferation [6,16], induced apoptosis of thymocytes and terminally differentiated T helper cells [31], downregulated the TCR ξ-chain [32], and upregulated CD25+ T regulatory cells [33]. To determine whether downregulation of the cell surface CD4 molecule by IDO has a role in the onset and regulation of the antigen-specific immune responses, two recombinant adenoviruses bearing either the EGFP (pAd-EGFP), a reporter gene, or the IDOEGFP gene (pAd-IDOEGFP) in which the gene of IDO was fused with the EGFP gene without a stop codon, were constructed in our laboratory, and a series of experiments for IDO immunomodulatory mechanisms were carried out. It was shown that when GFP coding gene is fused in frame with the target gene, the resulting polypeptide contains both fluorophore and enzymatic activity of the target protein. This GFP fusion protein can be easily detected by fluorescence microscopy or flow cytometry, thereby enabling the tagged proteins for gene regulation analysis [34]. For example, we fused YFP gene to integrase gene of HIV-1 and studied subcellular localization of this enzyme [35]. Of course, before determining whether IDO downregulates the cell surface CD4 molecule, we demonstrated that IDOEGFP protein inhibited the proliferation of C8166 cells infected with pAd-IDOEGFP (data not shown), thereby disclosing that the function of IDOEGFP protein is consistent with IDO protein.

By using MT-2 and C8166 cell lines that express the cell surface CD4 molecule, we showed for the first time that IDO downregulated the cell surface CD4 expression in these cells infected with pAd-IDOEGFP. Further analysis revealed that the downregulation of CD4 expression was partly due to the depletion of tryptophan. Because CD4 exerts a subtle influence on the natural status of immune responses, by influencing the sensitivity of antigen recognition and the precise nature of the response [36], these findings provide direct evidence showing that downregulation of CD4 is one of the mechanisms underlying IDO-mediated local immunosuppressive effect *in vivo*. Previous studies have suggested a strong correlation between IDO expression and the loss of T cell function in HIV-1-infected patients [10,16]. Some research demonstrated that HIV-1 inhibits CD4 + T cell proliferation by inducing IDO which might account for the impairment of T cell responses in these patients [10]. Our results hinted that IDO may impair T cell-mediated immune responses by downregulation of the cell surface CD4 expression. Although CD4 downregulation can be carried out by HIV-1 virus protein, such as HIV-1 Nef [37,38], it is interesting to discuss why cellular proteins, such as IDO, downregulate the cell surface CD4 molecule. Our results indicated that IDO induced downregulation of CD4 in MT-2 and C8166 cells. The most likely reason for the CD4 downregulation by IDO is the depletion of tryptophan. Tryptophan is the least available essential amino acid in the body, and T cell is susceptible in the absence of tryptophan [10,14,32]. Degradation of tryptophan by IDO might affect protein synthesis, and may probably downregulate the CD4 molecule on the T cell surface. Moreover, it has been well described that IDO expression can induce T cell apoptosis [5,6,9,10]. When there is a forced expression of IDO, it probably reduces the number of CD4 counts as the CD4 is the marker for T cells. In our experiments, both addition of tryptophan and 1-MT can partly restore downregulation of CD4 in pAd-IDOEGFP infected C8166 cells. Furthermore, our results demonstrate that downregulation of CD4 by IDO only occurred at the protein level, but not at the mRNA level, further implicating the possible role of tryptophan deletion in the regulation of CD4 expression. However, addition of tryptophan and 1-MT could not completely recover downregulated CD4 to the normal level in pAd-IDOEGFP infected C8166 cells, suggesting that besides depletion of tryptophan, other factors might also be involved in the downregulation of CD4. Therefore, it remains to be further investigated whether tryptophan metabolites induce downregulation of CD4, or CD4 downregulation requires the combined effects of tryptophan depletion and tryptophan catabolites. It has been proposed that HIV-1 Nef has the ability to downmodulate CD4 cell surface expression and that a leucine residue of the E160xxxLL motif in Nef is essential for the downregulation [39,40]. There is a E192xxxLL sequence in IDO and whether this motif is related to downmodulation of CD4 requires further study.

In conclusion, the data suggest a novel mechanism of immune regulation, of which IDO induces cell surface CD4 downregulation. Depletion of local tryptophan seems to be one of the reasons that IDO downregulates the expression of CD4. This observation strongly suggests that IDO may regulate T cell-mediated immune responses by downregulation of cell surface expression of the CD4 molecule.

## Figures and Tables

**Table 1:** CD4 copy-number analysis by real time PCR.

	β-actin Ct (Mean ± SD)	CD4 Ct (Mean ± SD)	2^−ΔΔCt^	GAPDH Ct (Mean ± SD)	CD4 Ct (Mean ± SD)	2^−ΔΔCt^
Control	30.35 ± 0.10	32.44 ± 2.63	1	31.34 ± 0.06	32.33 ± 2.51	1
pAd-EGFP	31.03 ± 0.46	33.58 ± 2.40	1.04 ± 0.05	32.34 ± 0.21	33.42 ± 2.55	1.02 ± 0.07
pAd-IDOEGFP	30.89 ± 0.27	33.02 ± 2.25	1.08 ± 0.09	31.86 ± 0.42	32.78 ± 2.10	1.05 ± 0.02

## Abbreviations

EGFP: enhanced green fluorescent protein
IDO: indoleamine 2,3-dioxygenase
MOI: multiplicity of infection
1-MT: 1-methyl-dl-tryptophan
pAd-EGFP: adenoviral vector carry EGFP, or recombinant adenovirus containing EGFP gene
pAd-IDOEGFP: adenoviral vector carrying IDOEGFP, or recombinant adenovirus containing IDOEGFP gene
